# Automated Pupillometry Is Able to Discriminate Patients with Acute Stroke from Healthy Subjects: An Observational, Cross-Sectional Study

**DOI:** 10.3390/brainsci14060616

**Published:** 2024-06-20

**Authors:** Irene Scala, Massimo Miccoli, Pia Clara Pafundi, Pier Andrea Rizzo, Francesca Vitali, Simone Bellavia, Jacopo Di Giovanni, Francesca Colò, Giacomo Della Marca, Valeria Guglielmi, Valerio Brunetti, Aldobrando Broccolini, Riccardo Di Iorio, Mauro Monforte, Paolo Calabresi, Giovanni Frisullo

**Affiliations:** 1 Department of Neuroscience, Catholic University of Sacred Heart, 00168 Rome, Italy; irene.scala92@gmail.com (I.S.); mmiccoli7@gmail.com (M.M.); pierandrearizzo01@gmail.com (P.A.R.); vitalifrancesca95@gmail.com (F.V.); bellavia.sim@gmail.com (S.B.); jacopodigiovanni@gmail.com (J.D.G.); colofra94@gmail.com (F.C.); giacomo.dellamarca@policlinicogemelli.it (G.D.M.); valerio.brunetti@policlinicogemelli.it (V.B.); aldobrando.broccolini@policlinicogemelli.it (A.B.); paolo.calabresi@policlinicogemelli.it (P.C.); 2 Dipartimento di Neuroscienze, Organi di Senso e Torace, Fondazione Policlinico Universitario A. Gemelli IRCCS, 00168 Rome, Italy; valeria.guglielmi@policlinicogemelli.it (V.G.); riccardo.diiorio@policlinicogemelli.it (R.D.I.); mauro.monforte@policlinicogemelli.it (M.M.); 3 Facility of Epidemiology and Biostatistics, Gemelli Generator, Fondazione Policlinico Universitario A. Gemelli IRCCS, 00168 Rome, Italy; piaclarapafundi88@gmail.com

**Keywords:** acute stroke, pupillary light reflex, automated pupillometry, NeurOptics, NPi, constriction velocity

## Abstract

Background: Automated pupillometry (AP) is a handheld, non-invasive tool that is able to assess pupillary light reflex dynamics and is useful for the detection of intracranial hypertension. Limited evidence is available on acute ischemic stroke (AIS) patients. The primary objective was to evaluate the ability of AP to discriminate AIS patients from healthy subjects (HS). Secondly, we aimed to compute a predictive score for AIS diagnosis based on clinical, demographic, and AP variables. Methods: We included 200 consecutive patients admitted to a comprehensive stroke center who underwent AP assessment through NPi-200 (NeurOptics^®^) within 72 h of stroke onset and 200 HS. The mean values of AP parameters and the absolute differences between the AP parameters of the two eyes were considered in the analyses. Predictors of stroke diagnosis were identified through univariate and multivariate logistic regressions; we then computed a nomogram based on each variable’s β coefficient. Finally, we developed a web app capable of displaying the probability of stroke diagnosis based on the predictive algorithm. Results: A high percentage of pupil constriction (CH, *p* < 0.001), a low constriction velocity (CV, *p* = 0.002), and high differences between these two parameters (*p* = 0.036 and *p* = 0.004, respectively) were independent predictors of AIS. The highest contribution in the predictive score was provided by CH, the Neurological Pupil Index, CV, and CV absolute difference, disclosing the important role of AP in the discrimination of stroke patients. Conclusions: The results of our study suggest that AP parameters, and in particular, those concerning pupillary constriction, may be useful for the early diagnosis of AIS.

## 1. Introduction

The pupillary light reflex (PLR), a highly conserved ancestral reflex, has provided an advantage for organisms to survive and thrive in different environments, providing the ability to adapt the pupil size and optimize visual performance under varying light conditions [1]. This subcortical, evolutionarily conserved reflex is mediated by both sympathetic and parasympathetic branches of the Autonomic Nervous System (ANS), whose first-order neurons are located in the midbrain and in the hypothalamus, respectively [2]. Despite PLR being conventionally considered a brainstem-mediated reflex, recent evidence suggests the presence of a cortical modulation in the PLR since several neurobehavioral syndromes [3,4] and cognitive processes [5] can influence the pupil’s reactivity to light.

In the clinical setting, the PLR assessment plays an important role in the non-invasive neuromonitoring of critically ill patients admitted to the Neurological Intensive Care Unit (N-ICU) [6] since alterations in its dynamics can detect intracranial hypertension early [7]. However, the manual assessment of pupil reactivity suffers from several limitations due to inter-rater variability and interferences from surrounding environmental conditions [8]. To overcome these limitations, automated devices capable of assessing PLR in a rapid, non-invasive, and operator-independent manner, namely Automated Pupillometers (APs), have been used in clinical practice in both clinical and research settings [9]. Growing evidence is, in fact, emerging on the role of AP in the prognostication of patients with traumatic brain injury [10] and after cardiac arrest [11]. Furthermore, due to its ability to detail the components of PLR in numerical variables, AP has been widely used in the diagnostic assessment of several diseases associated with an ANS imbalance [12,13,14] and for the evaluation of the efficacy of therapies acting on ANS [15].

To date, few data are available about the role of AP assessment in patients with acute stroke. Several studies focusing on the AP’s ability to detect stroke-related intracranial hypertension [16,17,18] neglected to analyze the mechanisms underlying the alteration of the PLR circuit, which can be involved due to ischemic damage. Furthermore, ischemic stroke, in the acute phase, is often associated with a cardiovascular autonomic imbalance [19], which is associated with a worse prognosis [20]. Due to these premises, we investigated, in acute ischemic stroke, the changes in the pupillary reactivity to light, detected through an AP assessment, which could become a useful and rapid tool for the early diagnosis of stroke in misleading cases.

The primary endpoint of this study is to define the ability of AP to discriminate patients with ischemic stroke during the acute phase (≤72 h from symptoms onset) from healthy subjects (HS). Secondly, our study aims to define a predictive score based on demographic, clinical, and pupillometric parameters, which could help clinicians in the early diagnosis of AIS.

## 2. Materials and Methods

### 2.1. Study Design and Population

In this single-center, observational, cross-sectional study, we enrolled consecutive adult patients admitted to the stroke unit of a comprehensive stroke center for a diagnosis of ischemic stroke and whose AP assessment was performed within 72 h of stroke onset. Exclusion criteria were brain hemorrhage, previous eye surgery, major eye trauma, or major eye diseases (e.g., glaucoma, cataracts requiring surgical intervention, severe retinopathy, optic neuritis), or neurological diseases affecting the ANS (e.g., Parkinsonism, autonomic neuropathy). To define the diagnostic ability of AP, we compared AIS patients (AIS group) with healthy subjects (HS group), choosing a 1:1 allocation ratio. Healthy subjects were recruited from patients’ relatives and other subjects who were at our hospital for non-medical reasons. The exclusion criteria for enrollment in the HS group were a recent diagnosis of AIS (e.g., in the three months preceding the AP assessment) and all the exclusion criteria adopted for patients with AIS (i.e., brain hemorrhage, previous eye surgery, major eye trauma, or major eye diseases, neurological diseases affecting the ANS).

All subjects were enrolled between March 2021 and February 2023. Written informed consent was obtained from study participants or their legal representatives. The study conformed to the principles of the 1964 Declaration of Helsinki and its later amendments. The research protocol was approved by the ethics committee of Comitato Etico of Fondazione Policlinico Universitario “A Gemelli” IRCCS—Rome (Study ID 5024/2022) on 6 October 2022. The study was conducted according to the Strengthening the Reporting of Observational Studies in Epidemiology (STROBE) guidelines.

### 2.2. Data Collection

Four trained investigators (I.S., M.M., P.A.R., and F.V.) performed the AP assessment in both eyes of all study participants using NPi-200^®^ (NeurOptics, Irvine, CA, USA), which is a hand-held device composed of an infrared camera able to repeatedly measure the pupil size without stimulating retinal receptors [21,22]. By delivering a calibrated light stimulus of fixed intensity and duration, the NPi-200^®^ induces PLR and then stores repetitive video images of the pupil at >30 frames per second for 3.2 s to decompose the brainstem reflex in numerical variables, which are quickly reported on a liquid crystal display [9,12].

All AP assessments were performed between 6:00 and 8:00 p.m. in order to minimize the impact of circadian rhythm and ambient lighting on PLR dynamics. Regarding HS, the AP assessment was performed at the same time and with the same procedures as stroke patients within an outpatient room. The assessments of PLR were repeated three consecutive times for each eye, considering, for each parameter, the mean value of the three measurements for the subsequent statistical analyses (“Overall value”) to minimize any recording errors. Finally, the mean parameters of the two eyes and their absolute differences were considered for further analysis. For the AIS group, the first AP assessment collected in the first 72 h after stroke onset was considered for the analysis as this time frame is widely regarded as the critical period that defines the acute phase of stroke [23]. For the HS group, AP evaluations were collected at the same patients’ conditions in an outpatient room. A summary of pupil parameters, their abbreviations, units of measurement, and meanings (Supplementary Table S1) and details about the functioning of NPi-200^®^ are available in the Supplementary Materials.

Clinical and demographic data were collected for each study participant through a medical record review for the AIS group and through an oral questionnaire for the HS group by two trained investigators (I.S., M.M.).

### 2.3. Statistical Analysis

Qualitative variables were expressed by absolute and relative percentage frequencies. Quantitative variables were reported as either the mean and standard deviation or median and interquartile range, as appropriate. Gaussian distribution was assessed by the Shapiro–Wilk test. Missing values were treated by the imputeR R package by multiple imputation with Lasso Regression methods centered on the mean for quantitative data, whilst classification trees for imputations by the “rpartC” function, centered on the mode, were applied to qualitative data [24].

There is no generally accepted approach for the estimation of the sample size for the derivation of score prediction models. Hence, we derived the score to include a number of covariates consistent with the rule of at least 10 events per candidate variable in the multivariate model, which is consistent with Transparent Reporting of a Multivariate Prediction Model for Individual Prognosis or Diagnosis (TRIPOD) guidelines [25].

Univariate and multivariate logistic regression models were performed to identify independent predictors of stroke for inclusion in the scoring system. Predictors to be included in the multivariate model were selected based on univariate analysis (*p* < 0.05 or suggestive, i.e., 0.05 ≤ *p* < 0.10).

The performance of the model was assessed based on diverse methods, such as Somers’ Dxy rank correlation, C-index, Nagelkerke R2 value, calibration intercept and slope, and Brier score [25]. The c-index can be interpreted as the area under the curve (AUC), namely a measure of accuracy in the model, where the value of one is indicative of the highest possible accuracy. Similarly, a Somers’ Dxy rank correlation (i.e., another discrimination index) of one is an index of perfectly discriminating predictions. Dxy has a simple relationship with c-index, i.e., Dxy = 2 × (c-0.5). “rms”, “magrittr”, and “predtools” R packages were used for the whole analyses set [26,27,28]. Finally, the Hosmer–Lemeshow goodness-of-fit test allowed for the calibration [29]. Calibration plots further provided a graphic representation of the association between the predicted and observed outcomes using locally weighted scatterplot smoothing [29]. The lateral axis shows the predicted probability of stroke for each patient, whereas the vertical axis shows the actual probability of stroke for each patient. It is ideal if the black line exactly coincides with the dotted line. The fit of the model was further evaluated using the fitting index RMSEA (Root Mean Square Error of Approximation), for which the best-fit values of the model is <0.05 [30]. The internal validation of the model was performed based on a bootstrap procedure with 1000 repetitions [29].

The performance of the model was assessed based on diverse methods, such as Somers’ Dxy rank correlation, C-index, Nagelkerke R2 value, calibration intercept and slope, and Brier score [25]. The Hosmer–Lemeshow goodness-of-fit test allowed for the calibration [29]. The fit of the model was further evaluated using the fitting index RMSEA (Root Mean Square Error of Approximation) [30]. Internal validation of the model was performed based on a bootstrap procedure with 1000 repetitions [29].

We then developed a scoring system, transforming the regression coefficients (β coefficients) of each variable into scores through appropriate mathematical transformations and plotting them into a nomogram as a predictive model tool [29]. The fitted model with the best performance was also used as the back end of an interactive web application that calculated the probability of the outcome based on the values of the predictors inserted by the user. This web app was developed and deployed using the Shiny framework for R [31]. Statistical significance was set at a *p*-value < 0.05. The whole statistical analysis set was performed with R software, version 4.3.0 (CRAN^®^, R Core 2023, Wien, Austria).

An extended explanation of the statistical analyses and details on sample size calculations are available in the Supplementary Materials.

## 3. Results

### 3.1. Characteristics of the Study Sample

Two hundred patients were included in the study [median age 73 years (IQR 61–81); 120 (60%) males]. The enrollment process is detailed in Supplementary Figure S1.

The median National Institute of Health Stroke Scale at admission was five (2–12). Fifty-five patients (27.5%) underwent intravenous thrombolysis, while mechanical thrombectomy was performed in 66 subjects (33.0%). Cardioembolism was the most represented pathogenetic cause in our population (31.5%), while in 51 patients (25.5%), the pathogenesis of stroke was not clarified at discharge. The left hemisphere was the one more frequently involved in the ischemic lesion (57.0%). Ischemic lesions were most frequently localized in frontal lobes (45.5%), followed by temporal ones (40.0%). A minority of patients presented an infra-tentorial lesion (Midbrain: 4.5%; Cerebellum: 11.5%). Seven patients (3.5%) presented brain edema at the follow-up neuroradiological examination. Characteristics of the AIS group are shown in detail in Supplementary Table S2.

Compared with the HS group, AIS patients were significantly older (*p* < 0.001), while both groups were predominantly male, without a significant difference between groups (male-to-female ratio 6:4). Cardiovascular risk factors were significantly more prevalent in the AIS group (all *p* < 0.001), as well as respiratory diseases (*p* = 0.002), and previous stroke (*p* < 0.001). Consequently, ACE inhibitors (*p* < 0.001), alpha- (*p* < 0.001) and beta-blockers (*p* < 0.001), sartans (*p* = 0.001), and calcium channel blockers (CCBs, *p* < 0.001) were more frequently taken by stroke patients than controls. Please refer to Table 1.

### 3.2. Descriptive Analysis of Pupillometric Parameters

Stroke patients presented a significantly higher median overall Neurological Pupil Index (NPi) and higher mean Percentage of Constriction (CH) compared to HS (both *p* < 0.001). Conversely, the median overall Baseline Pupil Diameter (BPD) (*p* = 0.028), Minimum Pupil Diameter (MIN) (*p* < 0.001), and Mean Average Constriction Velocity (CV) (*p* = 0.003) were significantly lower in the HS group. No significant differences were observed in other overall pupillometric parameters. Noteworthy, absolute differences between the pupillary parameters of the two eyes were significantly higher in the AIS group for all considered variables (*p* < 0.001) (Table 2).

### 3.3. Potential Predictors of Stroke

In the univariate analysis, older age was found to be significantly associated with a higher risk of stroke (*p* < 0.001). Among comorbidities, previous stroke (*p* < 0.001), hypertension (*p* < 0.001), and atrial fibrillation (*p* < 0.001) were the strongest predictors of stroke diagnosis. Also, diabetes (*p* < 0.001), dyslipidemia (*p* < 0.001), and respiratory diseases (*p* = 0.002) obtained a high OR, ranging between 2.60 and 4.0. All anti-hypertensive pharmacological treatments were significantly associated with a greater risk of stroke (*p* < 0.001).

Overall, a higher NPi disclosed only a suggestive association with stroke risk (OR 1.58, 95%CI 0.94–2.67; *p* = 0.082). A small MIN and a slow CV instead were significantly associated with stroke diagnosis (OR 0.45, 95%CI 0.28–0.71; *p* = 0.001 and OR 0.64, 95%CI 0.47–0.86; *p* = 0.003, respectively). Moreover, a higher CH disclosed a significant association with stroke (OR 1.04, 95%CI 1.01–1.08; *p* = 0.007). Concerning absolute differences among the pupillary parameters between the two eyes, higher values were found to be strongly indicative of stroke diagnosis regarding BPD (OR 6.57, 95%CI 2.85–15.14; *p* < 0.001), CH (OR 1.27, 95%CI 1.18–1.38; *p* < 0.001), CV (OR 4.97, 95%CI 2.30–10.71; *p* < 0.001), and MCV (OR 3.51, 95%CI 2.05–6.01; *p* < 0.001). Absolute NPi, MIN, LAT, and DV differences, though statistically significant, presented a too-high confidence interval for consideration as reliable findings (see Table 3).

### 3.4. Predictive Performance

We computed several models of multivariate logistic regression to find the one with the best accuracy to predict stroke diagnosis. Details about the selection pathway of variables to be included in the predictive score are available in the Supplementary Materials.

**Figure 1:**
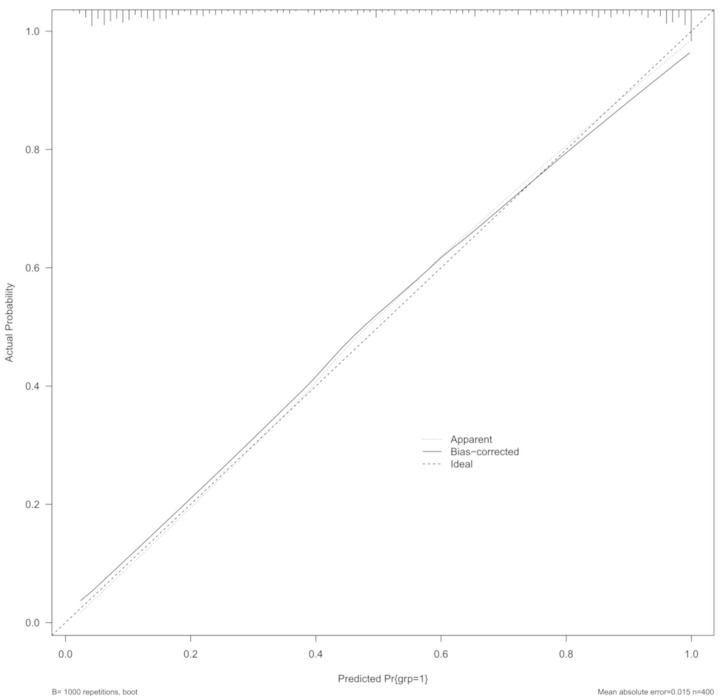
Calibration plot of the final model using bootstrap internal validity resampling method, randomly sampling 100 returnable cases. The lateral axis shows the predicted probability of stroke for each patient, while the vertical axis shows the actual probability of stroke for each patient. It is ideal if the straight line exactly coincides with the dotted line.

The third model of our selection process disclosed an overall excellent fit (C-index 0.903) with a corrected Dxy of 0.776, which is very close to the unadjusted of 0.806, and an R2 correlation near 0.6 (0.598). The calibration plot using bootstrap internal validity resampling provided confirmation of the stability of the model, with an MAE of 0.015 and an RMSE of 0.00030 with the deviation of the calibration curve, which is quite small (see Figure 1).

Such a model disclosed the following as independent predictors of stroke diagnosis: advanced age (*p* < 0.001), previous stroke (*p* = 0.001) and atrial fibrillation (*p* = 0.010) among clinical data, ACE inhibitors (*p* < 0.001), and CCBs (*p* = 0.033) among concomitant therapies. Pupillometry data instead showed a high overall mean CH (OR 1.21, 95%CI 1.11–1.33; *p* < 0.001), a low CV (OR 0.26, 95%CI 0.11–0.61; *p* = 0.002), and high CH and CV absolute differences (OR 1.13, 95%CI 1.01–1.26; *p* = 0.036 and OR 4.75, 95%CI 1.64–13.73; *p* = 0.004, respectively) to be independent predictors of stroke (Supplementary Table S3, Table 4).

Based on this final model, we constructed the related nomogram to define a score for predicting stroke diagnosis inclusive of pupillometry parameters. Considering the total score, given by the sum of the points obtained from each variable included in the nomogram, we observed that the probability of stroke occurrence overcame 90% with a total score ≥ 17. An estimate of the probability of the outcome can also be interactively obtained using the web app developed on top of the final model (https://strokeunitgemelli.shinyapps.io/stroke_prediction_pupillometry/, accessed on 23 September 2023).

**Figure 2:**
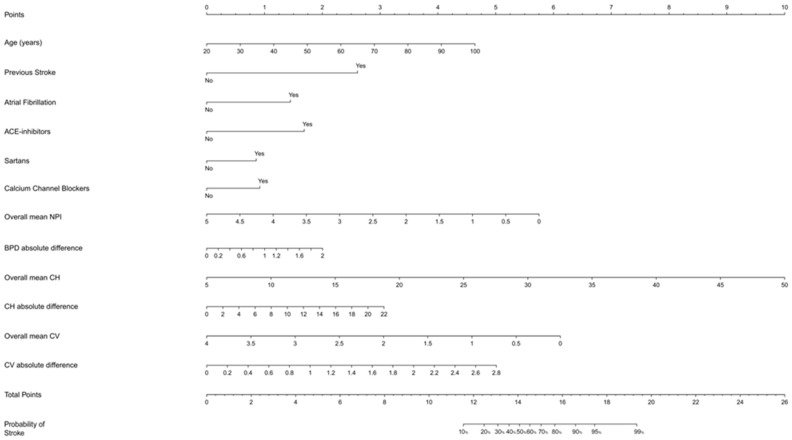
Nomogram displaying the probability of the occurrence of stroke. The upper points help assign the correct score to each variable, whilst the total points in the bottom part of the nomogram, alongside the predicted probability in the last line, allow the assignment of the predicted probability of stroke according to the total score. Abbreviations: NPi, Neurological Pupil Index; BPD, Baseline Pupil Diameter; CH, Percentage of Constriction; CV, Average Constriction Velocity.

With regard to specific parameters (Figure 2), we found that the highest contribution in the stroke diagnosis prediction is provided by an overall mean CH, followed by an overall mean NPi, mean CV, and CV absolute difference, disclosing the important role of pupillometry in the discrimination of stroke. On the contrary, clinical and demographic parameters played a minor role in the diagnostic predictivity of stroke, contributing globally with about 12 points compared to more than 10 points for a single PA parameter (overall CH).

## 4. Discussion

In our study, many AP parameters differed between patients with AIS and HS. Furthermore, several components of the PLR, and, in particular, those concerning pupillary constriction, were found to be independent predictors of acute stroke diagnosis. Analyzing our scoring system, we found that the AP parameters, such as high overall CH, low CV, and a reduction in overall pupil reactivity (NPi), accounted heavily on the calculation of the predictive score. Furthermore, a large absolute difference in these parameters (CH and CV) between the two eyes was also an independent predictor of stroke diagnosis, suggesting that ischemic injury led to lateralized impairment of the PLR dynamics.

To date, no studies have analyzed the ability of AP to discriminate AIS patients from HS. The limited evidence focusing on patients with AIS confirmed that AP can detect sudden clinical/radiological worsening early, suggestive of intracranial hypertension in such populations [17,18,32,33]. A reduction in mean overall CV, DV, and CH [17], or at least one NPi < 3 [32], predicted the development of radiological/clinical evidence of brain edema in patients who underwent mechanical thrombectomy. Similarly, at least one NPi value < 2.8 was able to predict neurological worsening in patients with large hemispheric ischemic/hemorrhagic strokes of the anterior circulation [33]. However, these studies, recruiting only patients with large hemispheric strokes of the anterior circulation admitted to the N-ICU, selected a more severe patient population than our study population, which is a realistic cross-section of AIS patients admitted to a sub-intensive ward.

In addition, many of these studies included patients who underwent mechanical thrombectomy after general anesthesia or sedation, capable of altering PLR dynamics per se [34]. Finally, no comparisons were made with the non-stroke population, making it impossible to define whether the alterations in pupillary dynamics were only a consequence of intracranial hypertension or the stroke itself. Since only 3.5% of our population presented signs of brain edema, the results of our study support the latter hypothesis.

In apparent contrast to previous evidence suggesting that a reduced pupillary reactivity is univocally linked to pathological conditions [35], we found that subjects of the AIS group had a higher overall NPi than healthy subjects and, in the univariate logistic regression, NPi was found to be only a suggestive predictor of AIS. Instead, in the multivariate logistic regression model, the NPi OR reversed (i.e., OR < 1), suggesting that a high pupil reactivity is a “protective factor” for stroke diagnosis, although this did not reach statistical significance as an independent predictor of stroke. These results are in line with a recent study that reported that NPi failed to be an independent predictor of delayed cerebral ischemia in patients admitted to the N-ICU for subarachnoid hemorrhage [36]. These data could be explained considering that NPi is influenced almost exclusively by the brainstem function, making it a useful measure for the early detection of intracranial hypertension [18,37], while it is only marginally influenced by cortical activity, contrary to other AP variables [38].

Although an understanding of physiological processes underlying altered pupil reactivity is beyond the scope of our study, we can assume that a major role may be played by the dysregulation of the descending activating cortical inputs directed towards the locus coeruleus, a pontine, noradrenergic nucleus which is the cerebral structure mostly involved in the regulation of the emotional and cognitive control of the pupil diameter [39]. This nucleus receives diffuse inputs from the cerebral cortex [40], and is directly involved in the potentiation of the sympathetic outflow to the iris and in the inhibition of the Edinger–Westphal nucleus, leading to pupil dilation and a reduction in the excursion of pupil diameter after exposure to a light stimulus [41]. Consequently, an impairment in such cortical, excitatory pathways due to ischemic brain injuries may reduce the activation of locus coeruleus and, consequently, reduce the inhibition of PLR, increasing CH, as in our study. Furthermore, the impairment in locus coeruleus activity may reduce the BPD, such as in our stroke population, and consequently, reduce CV since these two parameters are strongly correlated [38]. Finally, the increase in the absolute differences of CV and CH of the two eyes suggests a strong lateralization of the stroke-induced imbalance of PLR cortical modulation.

To summarize the evidence emerging from our study, we can therefore state that the pupillary alterations found in our population of AIS patients could be attributed to the dysfunction of the locus coeruleus (and therefore of the sympathetic nervous system) derived from a reduced descending cortical stimulation resulting from the cerebral ischemia. The dysfunction of the sympathetic regulation of the PLR could, therefore, induce disproportionate parasympathetic hypertonicity, which then leads to an accentuation of the pupillary reflex to light, as found in our patients with acute stroke, in whom CH was significantly increased.

This is the first study that directly compared AP-collected PLR parameters between subjects with AIS and HS, providing evidence that AIS is a condition leading to a complex imbalance of the descending modulation of the pupillary dynamics. Furthermore, PLR parameters were found to be the strongest predictors of stroke diagnosis, even when adjusted for cardiovascular risk factors, suggesting that alterations of pupillary dynamics are closely related to acute stroke. The results of our study suggest that AP could be used as a diagnostic tool for the early recognition of ischemic stroke in doubtful cases, helping clinicians in the process of differential diagnosis. In addition, the automated evaluation of the PLR may also be useful for the detection of acute ischemic stroke-related autonomic dysfunction, even with the association of other diagnostic tools, such as the contemporary analysis of heart rate variability.

Furthermore, the findings of our study lay the foundations for other potential future applications of AP in the clinical setting of patients with AIS. Future studies may investigate whether acute-phase AP might be able to discriminate AIS patients from patients with stroke-mimicking conditions in the emergency department to aid the clinician in the difficult decision-making process of patients with negative baseline brain CT scans. Furthermore, the role of AP in predicting stroke prognosis should also be investigated, both in the setting of stroke units and rehabilitation units, in order to rapidly identify patients with an autonomic imbalance who could benefit from close clinical and instrumental monitoring.

Our study has several limitations. First, there is the cross-sectional design. Secondly, the environmental conditions in which AP assessments were performed included AIS patients evaluated in a sub-intensive care unit, while healthy controls were in an outpatient room. Furthermore, ambient lighting conditions were not controlled, possibly leading to recording errors. It should, however, be considered that the NeurOptics devices were designed to reduce interference of ambient lighting and other environmental conditions on the PLR dynamics [9] and that our recording conditions were in line with clinical practice. In addition, previous studies have verified the reliability of AP assessments performed in the context of stroke units [42]. Moreover, we did not perform an external validation of our web abb for the discrimination of AIS patients and HS, limiting the external validity of our study to different stroke patient populations. Finally, we compared nonhomogeneous groups. To address this shortcoming, we ran multiple multivariate logistic regression models to find independent predictors of stroke diagnosis.

## 5. Conclusions

In conclusion, the results of our study suggest that AIS leads to a complex alteration of the descending pathways that modulate PLR dynamics. Consequently, AP may be a simple and easy-to-use tool to assist the clinician in the early diagnosis of stroke, especially in tricky cases. Due to the limitations of our study and the absence of a comparison between patients with AIS and subjects with stroke-mimicking conditions, further longitudinal, multicenter studies are needed.

## Figures and Tables

**Table 1:** General characteristics of the study sample.

	AIS Group	HS Group	*p*
	(n = 200)	(n = 200)	
**Demographics**			
Age (years)	73 (61–81)	58 (50–67)	**<0.001**
Sex (male)	120 (60)	116 (58)	0.760
**Comorbidities**			
Diabetes	48 (24)	15 (7.5)	**<0.001**
Hypertension	156 (78)	54 (27)	**<0.001**
Dyslipidemia	86 (43)	45 (22.5)	**<0.001**
Previous stroke	42 (21)	4 (2)	**<0.001**
Atrial fibrillation	50 (25)	8 (4)	**<0.001**
Cancer	32 (16)	28 (14)	0.675
Hepatopathy	7 (3.5)	7 (3.5)	1.000
Respiratory disease	29 (14.5)	10 (5)	**0.002**
Obesity	33 (16.5)	41 (20.5)	0.367
**Pharmacological data**			
Beta blockers	87 (43.5)	22 (11)	**<0.001**
Alpha blockers	37 (18.5)	9 (4.5)	**<0.001**
ACE inhibitors	103 (51.5)	30 (15)	**<0.001**
Sartans	48 (24)	22 (11)	**0.001**
Calcium channel blockers	74 (37)	17 (8.5)	**<0.001**
Antidepressants	12 (6)	5 (2.5)	0.135

Abbreviations: AIS, acute ischemic stroke; HS, healthy subjects Qualitative variables are expressed as absolute and relative percentage frequencies; quantitative data are presented as median and interquartile ranges.

**Table 2:** Distribution of pupillary parameters in both study groups.

	AIS Group	HS Group	*p*
	(n = 200)	(n = 200)	
**Pupillometry parameters**			
**NPi**			
Overall	4.50 (4.25–4.70)	4.36 (4.17–4.53)	**<0.001**
Absolute difference	0.10 (0.10–0.30)	0.10 (0.03–0.14)	**<0.001**
**Baseline Pupil Diameter (mm)**			
Overall	3.34 (2.80–3.87)	3.50 (3.14–3.93)	**0.028**
Absolute difference	0.29 (0.12–0.53)	0.20 (0.10–0.35)	**<0.001**
**Minimum Pupil Diameter (mm)**			
Overall	2.39 (2.01–2.66)	2.54 (2.31–2.78)	**<0.001**
Absolute difference	0.16 (0.07–0.31)	0.09 (0.04–0.17)	**<0.001**
**Percentage of Constriction (%)**			
Overall	29.18 (7.16)	27.41 (5.67)	**0.006**
Absolute difference	4.0 (2.0–8.0)	2.0 (1.0–4.0)	**<0.001**
**Average Constriction Velocity (mm/s)**			
Overall	1.98 (0.71)	2.11 (0.62)	**0.003**
Absolute difference	0.32 (0.15–0.58)	0.21 (0.10–0.42)	**<0.001**
**Maximum Constriction Velocity (mm/s)**			
Overall	3.03 (2.32–3.61)	3.01 (2.50–3.65)	0.313
Absolute difference	0.50 (0.19–0.80)	0.28 (0.14–0.56)	**<0.001**
**Reflex Latency (s)**			
Overall	0.23 (0.21–0.27)	0.24 (0.22–0.26)	0.493
Absolute difference	0.03 (0.00–0.04)	0.01 (0.01–0.02)	**<0.001**
**Dilation Velocity (mm/s)**			
Overall	0.91 (0.29)	0.89 (0.23)	0.651
Absolute difference	0.15 (0.07–0.26)	0.10 (0.04–0.18)	**<0.001**

Abbreviations: AIS, acute ischemic stroke; HS, healthy subjects; NPi, Neurological Pupil Index. Quantitative data are presented as either mean and standard deviation (SD) or median and interquartile range (IQR), as appropriate.

**Table 3:** Results of the univariate logistic regression.

	OR (95%CI)	*p*
**Demographics**		
Age	1.07 (1.05;1.09)	**<0.001**
Male Sex	1.09 (0.73; 1.62)	0.684
**Comorbidities**		
Diabetes	3.89 (2.10;7.23)	**<0.001**
Hypertension	9.59 (6.07;15.15)	**<0.001**
Dyslipidemia	2.60 (1.68;4.01)	**<0.001**
Previous stroke	13.0 (4.57;37.09)	**<0.001**
Atrial fibrillation	8.0 (3.68;17.39)	**<0.001**
Cancer	1.17 (0.67;2.03)	0.576
Hepatopathy	1.00 (0.34;2.90)	1.000
Respiratory disease	3.22 (1.52;6.81)	**0.002**
Obesity	0.77 (0.46;1.27)	0.304
**Concomitant Medications**		
Beta blockers	6.23 (3.69;10.52)	**<0.001**
Alpha blockers	4.82 (2.26;10.28)	**<0.001**
ACE inhibitors	6.02 (3.73;9.69)	**<0.001**
Sartans	2.55 (1.48;4.42)	**0.001**
Calcium channel blockers	6.32 (3.56;11.22)	**<0.001**
**Pupillometry parameters**		
**NPi**		
Overall	1.58 (0.94;2.67)	0.082
Absolute difference	114.13 (19.77;658.66)	**<0.001**
**Baseline Pupil Diameter**		
Overall	0.80 (0.61;1.06)	0.121
Absolute difference	6.57 (2.85;15.14)	**<0.001**
**Minimum Pupil Diameter**		
Overall	0.45 (0.28;0.71)	**0.001**
Absolute difference	56.39 (12.07;263.43)	**<0.001**
**Percentage of Constriction**		
Overall	1.04 (1.01;1.08)	**0.007**
Absolute difference	1.27 (1.18;1.38)	**<0.001**
**Average Constriction Velocity**		
Overall	0.64 (0.47;0.86)	**0.003**
Absolute difference	4.97 (2.30;10.71)	**<0.001**
**Maximum Constriction Velocity**		
Overall	0.90 (0.73;1.10)	0.313
Absolute difference	3.51 (2.05;6.01)	**<0.001**
**Reflex Latency**		
Overall	0.07 (0.00;31.38)	0.389
Absolute difference	Inf^ (Inf^;Inf^)	**<0.001**
**Dilation Velocity**		
Overall	1.19 (0.57;2.49)	0.650
Absolute difference	65.71 (10.23;421.95)	**<0.001**

Abbreviations: OR, odds ratio; CI, confidence interval.

**Table 4:** Results of the third model of multivariate logistic regression.

	Model 3
	OR (95%CI); *p*
**Demographics**	
Age	**1.05 (1.03;1.08); <0.001**
**Comorbidities**	
Previous stroke	**9.70 (2.69;34.92); 0.001**
Atrial fibrillation	**3.53 (1.36;9.17); 0.010**
**Concomitant medications**	
ACE inhibitors	**4.34 (2.27;8.32); <0.001**
Sartans	2.11 (0.98;4.53); 0.056
CCBs	**2.23 (1.06;4.65); 0.033**
**Pupillometry parameters**	
Overall NPi	0.37 (0.10;1.28); 0.116
BPD absolute diff.	2.39 (0.73;7.88); 0.151
Overall CH	**1.21 (1.11;1.33); <0.001**
CH absolute diff.	**1.13 (1.01;1.26); 0.036**
Overall CV	**0.26 (0.11;0.61); 0.002**
CV absolute diff.	**4.75 (1.64;13.73); 0.004**

Abbreviations: OR, odds ratio; CI, confidence interval; CCBs, calcium channel blockers; NPi, Neurological Pupil Index; BPD, Baseline Pupil Diameter; CH, Percentage of Constriction; CV, Average Constriction Velocity.

## Data Availability

The raw data of this study are available, upon reasonable request, from the corresponding author of the study. The data are not publicly available due to privacy restrictions in accordance with the Ethics Committee and Data Protection Office of our Institute.

## Supplementary Materials

The following supporting information can be downloaded at: https://www.mdpi.com/article/10.3390/brainsci14060616/s1, Figure S1: The flow diagram of the study; Figure S2: The calibration curve of the first two outcome prediction models; Figure S3: The nomogram of the first outcome prediction model; Figure S4: The nomogram of the second outcome prediction model; Table S1: A descriptive table reassuming the parameters collected by NPi-200^®^ (NeurOptics, Irvine, CA, USA); Table S2: A summary of the clinical and radiological characteristics of the AIS group; Table S3: All three multivariate logistic regression models tested.
