# Supplementary material for: Automated Pupillometry Is Able to Discriminate Patients with Acute Stroke from Healthy Subjects: An Observational, Cross-Sectional Study
Source: Brain Sci. 2024 Jun 20;14(6):616. doi: 10.3390/brainsci14060616 (PMC11202086; doi:10.3390/brainsci14060616)
Supplement: Supplementary file 1 [file brainsci-14-00616-s001.zip › Supplementary Figure Legends.pdf]

Supplementary Figures Legend

Supplementary Figure S1. Calibration plot of the first model (Panel A) and of the second model (Panel B) using bootstrap internal validity resampling method, randomly sampling 100 returnable cases. The lateral axis shows the predicted probability of stroke for each patient, while the vertical axis shows the actual probability of stroke for each patient. It is ideal if the straight line exactly coincides with the dotted line.

Supplementary Figure S2. Nomogram related to the first model, displaying the probability of the occurrence of stroke. The upper points help assign the correct score to each variable, whilst total points in the bottom part of the nomogram, alongside the predicted probability in the last line, allow the assignment of the predicted probability of stroke according to the total score. Abbreviations: NPi, Neurological Pupil Index; BPD, Baseline pupil diameter; MIN, minimum; CH, Percentage of Constriction; (M)CV: (Maximum) Constriction Velocity.

Supplementary Figure S3. Nomogram related to the second model, displaying the probability of the occurrence of stroke. The upper points help assign the correct score to each variable, whilst total points in the bottom part of the nomogram, alongside the predicted probability in the last line, allow the assignment of the predicted probability of stroke according to the total score. Abbreviations: NPi, Neurological Pupil Index; BPD, Baseline pupil diameter; MIN, Minimum pupil diameter; CH, Percentage of Constriction; CV, Constriction Velocity

Supplementary Figure S4. The nomogram of the third outcome prediction model.
