# Supplementary material for: Automated Pupillometry Is Able to Discriminate Patients with Acute Stroke from Healthy Subjects: An Observational, Cross-Sectional Study
Source: Brain Sci. 2024 Jun 20;14(6):616. doi: 10.3390/brainsci14060616 (PMC11202086; doi:10.3390/brainsci14060616)
Supplement: Supplementary file 1 [file brainsci-14-00616-s001.zip › Supplementary methods.pdf]

Supplementary methods

Automated pupillometry functioning

NPi-200® (NeurOptics, Irvine, CA, USA) is a hand-held device composed of an infrared camera able to repeatedly measure the pupil size without stimulating retinal receptors [1,2]. By delivering a calibrated light stimulus of fixed intensity and duration, the NPi-200® induces PLR and then stores repetitive video images of the pupil at >30 frames per second for 3.2 s to decompose the brainstem reflex in eight numerical variables, which are quickly reported on a liquid crystal display [3,4]. Furthermore, the device provides the examiner with an overall summary of pupil reactivity, the Neurological Pupil index (NPi), a scalar value ranging from 0 to 4.9. An NPi ≥ 3 is indicative of a normal pupil response to light, while values below this cut-off are suggestive of a "sluggish pupil" [4].

Statistical analysis

The study sample was described in its sociodemographic, clinical, and AP data by appropriate descriptive statistics indices. In depth, qualitative variables were expressed by absolute and relative percentage frequencies. Quantitative variables, indeed, were reported as either mean and standard deviation (SD) or median and interquartile range (IQR), accordingly. Gaussian distribution was assessed by the Shapiro Wilk test. Missing values were treated by imputeR R package, by multiple imputation with Lasso Regression methods centered on the mean for quantitative data, whilst classification trees for imputation by "rpartC" function, centered on the mode, were applied to qualitative data [5]. There is no generally accepted approach for the estimation of the sample size for derivation of score prediction models. Hence, we based for the derivation of the score to include in the multivariate model a number of covariates consistent with the rule of at least 10 events per candidate variable, consistent with Transparent Reporting of a multivariate prediction model for Individual Prognosis or Diagnosis (TRIPOD) guidelines [6].

Univariate and multivariate logistic regression models were performed to identify independent predictors of stroke to be included in the scoring system. In depth, we computed Odd Ratios (ORs) and 95% Confidence Intervals (CIs) of the predictor candidates for the outcome by univariate logistic regression models. Predictors to be included in the multivariate model were selected based on univariate analysis ($p<0.05$ or suggestive, i.e. $0.05 \leq p<0.10$). The multivariate logistic regression models produced a β coefficient and a Standard Error (SE) for each variable. The performance of the model was assessed based on diverse methods, such as Somers' Dxy rank correlation, C-index, Nagelkerke R2 value, calibration intercept and slope, and Brier score [6]. The c-index can be interpreted as an area-under-the-curve (AUC), namely a measure of accuracy of the model, where the value of 1 is indicative of the highest possible accuracy. Similarly, a Somers' Dxy rank correlation (i.e. another discrimination index) of 1 is index of perfectly discriminating predictions. Dxy has a

simple relationship with c-index, i.e. Dxy = 2×(c-0.5). “rms”, “magrittr” and “predtools” R packages were used for the whole analyses set [7-9]. Finally, the Hosmer-Lemeshow goodness-of-fit test allowed for the calibration [10]. Calibration plots further provided a graphic representation of the association between the predicted and observed outcomes by locally weighted scatterplot smoothing [10]. Lateral axis shows the predicted probability of stroke of each patient, while the vertical axis shows the actual probability of stroke of each patient. It is ideal if the black line exactly coincides with the dotted line. The fit of the model was further evaluated using the fitting index RMSEA (Root Mean Square Error of Approximation), for which the best fit values of the model is < 0.05 [11]. Internal validation of the model was performed based on a bootstrap procedure with 1000 repetitions [10].

We then passed to develop a scoring system to predict the diagnosis of AIS providing an integer value to each predictor included in the scoring system based on each variable’s β coefficient in our sample [10]. In depth, the regression coefficients of the model are transformed into scores through appropriate mathematical transformations and plotted into a nomogram as predictive model tool [10]. For each independent variable, a straight line perpendicular to the Points axis (through a ruler) is made at that point, and the intersection point represents the score under the value of the independent variable. The corresponding points of these independent variables of each patient can be calculated in total. We can get total points, which will trace to the outcome probability axis with a perpendicular line.

The fitted model with the best performances was also used as back-end of an interactive web application that calculates the probability of the outcome based on the values of the predictors inserted by the user. This web-app has been developed and deployed using the Shiny framework for R [12].

Statistical significance was set at a p-value < 0.05. Suggestive p-values were further reported ($0.05 \leq p < 0.10$). The whole statistical analysis set was performed with R software, version 4.3.0 (CRAN ®, R Core 2023, Wien, Austria).

Sample size calculation

Up to our knowledge, no study has investigated the creation of a potential score to discriminate stroke patients according to pupillometry data. Hence, this represents a first attempt on a single-center basis. Based on the study design, which pertains the creation of a predictive model and a potential score, which alongside implies the use of regression methods, we included 400 individuals, equally split into stroke patients and controls without stroke.

References

1. Larson, M.D.; Behrends, M. Portable infrared pupillometry: a review. *Anesth Analg* **2015**, *120*, 1242-1253, doi:10.1213/ANE.0000000000000314.

2. Lussier, B.L.; Olson, D.M.; Aiyagari, V. Automated Pupillometry in Neurocritical Care: Research and Practice. *Curr Neurol Neurosci Rep* **2019**, *19*, 71, doi:10.1007/s11910-019-0994-z.
3. Bellavia, S.; Scala, I.; Luigetti, M.; Brunetti, V.; Gabrielli, M.; Zileri Dal Verme, L.; Servidei, S.; Calabresi, P.; Frisullo, G.; Della Marca, G. Instrumental Evaluation of COVID-19 Related Dysautonomia in Non-Critically-Ill Patients: An Observational, Cross-Sectional Study. *J Clin Med* **2021**, *10*, doi:10.3390/jcm10245861.
4. NeurOptics. NeurOptics—The Leader in the Science of Pupillometry. Available online: https://neuroptics.com. (accessed on 9 May 2023).
5. Steffen Moritz, L.F., Gen Nowak, Alan. H. Welsh, Terry. J. O'Neill. _imputeR: A General Multivariate Imputation Framework_. *R package version 2.2, <https://CRAN.R-project.org/package=imputeR>* **2020**.
6. Moons, K.G.; Altman, D.G.; Reitsma, J.B.; Ioannidis, J.P.; Macaskill, P.; Steyerberg, E.W.; Vickers, A.J.; Ransohoff, D.F.; Collins, G.S. Transparent Reporting of a multivariable prediction model for Individual Prognosis or Diagnosis (TRIPOD): explanation and elaboration. *Ann Intern Med* **2015**, *162*, W1-73, doi:10.7326/M14-0698.
7. Frank E Harrell Jr. _rms: Regression Modeling Strategies_ *R package version 6.3-0, <https://CRAN.R-project.org/package=rms>* **2022**.
8. Bache S, W.H. _magrittr: A Forward-Pipe Operator for R_. *R package version 2.0.3, <https://CRAN.R-project.org/package=magrittr>.* **2022**.
9. Sadatsafavi M, S.A. _predtools: Prediction Model Tools_. *R package version 0.0.2, <https://CRAN.R-project.org/package=predtools>.* **2021**.
10. Steyerberg EW. *Clinical Prediction Models: A Practical Approach to Development, Validation, And Updating*, 2nd edition ed.; Springer: 2009.
11. Hu LT, B.P. Cutoff Criteria for Fit Indexes in Covariance Structure Analysis: Conventional Criteria versus New Alternatives. *Structural Equation Modeling: A Multidisciplinary Journal* **1999**, *6*, 1-55, doi:https://doi.org/10.1080/10705519909540118.
12. Chang W, C.J., Allaire J, Sievert C, Schloerke B, Xie Y, Allen J, McPherson J, Dipert A, Borges B. shiny: Web Application Framework for R. . *<https://shiny.posit.co/, https://github.com/rstudio/shiny.>* **2023**.
