# Supplementary material for: Automated Pupillometry Is Able to Discriminate Patients with Acute Stroke from Healthy Subjects: An Observational, Cross-Sectional Study
Source: Brain Sci. 2024 Jun 20;14(6):616. doi: 10.3390/brainsci14060616 (PMC11202086; doi:10.3390/brainsci14060616)
Supplement: Supplementary file 1 [file brainsci-14-00616-s001.zip › Supplementary results.pdf]

Supplementary Results

Predictive Performance

The first model was constructed with all the covariates which resulted to be significant/suggestive predictors of stroke in the univariate analysis. Among the demographic and clinical/pharmacological parameters, an advanced age (OR 1.04, 95%CI 1.0.2-1.07; p=0.001), a clinical history of previous stroke (OR 9.05, 95%CI 2.48-32.95; p=0.001) and atrial fibrillation (OR 2.96, 95%CI 1.04-8.41; p=0.042), and ACE-inhibitors intake (OR 3.28, 95%CI 3.28-7.68; p=0.006) were all significantly associated with a higher risk of being diagnosed with a stroke. Pupillometry data instead showed as independent predictors of stroke a high overall mean CH (OR 1.21, 95%CI 1.09-1.34; p<0.001) and a slow CV (OR 0.30, 95%CI 0.12-0.75; p=0.010), and a suggestive role for high CH, BPD and CV absolute differences (OR 1.13, 95%CI 1.00-1.27; p=0.051, OR 3.10, 95%CI 0.83-11.49; p=0.091, and OR 3.90, 95%CI 0.94-16.14; p=0.060, respectively) (**Table S3**). Once fitted the model, we looked up at its stability and accuracy by the analysis of diverse discrimination indexes. The accuracy of this first model, though an excellent C-index (C = 0.906), must be analyzed alongside the other fitting indices. The Brier score is a strictly proper scoring rule and can be decomposed into discrimination and calibration components (the lower the score, the better the predictions). In our case a ***Brier score*** = 0.122, confirms a good accuracy. However, the strict relationship between C-index and Somers' Dxy rank correlation and its value of 0.812 decrease till 0.761 when corrected after bootstrap calibration procedure with 1000 replicates, thus indicating a quite overoptimistic model, in which more likely several variables can be ruled out due to a poor contribution. Optimism denotes the amount of estimated overestimation by the model. The corrected ***Dxy*** is the original estimate minus the optimism. In this case, the bias-corrected ***Dxy*** is much smaller than the original. The further calibration curve using bootstrap internal validity resampling method, and the relative plot provides some evidence that our models is overfitting: the model overestimates low probabilities and underestimates high probabilities. In depth, the calibration plot provided us a mean absolute error (MAE) of 0.027, a mean squared error (RMSE) of 0.00088 (see **Figure S2A**). Moreover, the calibration curve visualizes the results of Hosmer-Lemeshow fit goodness test. The closer to Y = X the prediction rate and the actual occurrence rate are, with p value of Hosmer-Lemeshow goodness-of-fit test greater than 0.05, the better the model is calibrated. In our case, the Calibration curve deviates, indicating that the model must be re-evaluated. Then, the coefficients of the regression model were transformed into scores through appropriate mathematical transformations and plotted as a nomogram as a predictive model tool of the probability of stroke diagnosis (see **Figure S3**). The nomogram clearly disclose that the contribution of several parameters is negligible (e.g., diabetes, hypertension, dyslipidemia, alpha and beta-blockers, and MCV absolute difference).

We thus fitted a second model, erasing the aforementioned predictors. Such model disclosed as independent predictors of stroke diagnosis: advanced age (OR 1.05, 95%CI 1.0.2-1.07; p<0.001), a previous stroke (OR 10.02, 95%CI 2.76-36.41;

p<0.001) and atrial fibrillation (OR 3.37, 95%CI 1.28-8.87; p=0.014) among clinical data, and ACE-inhibitors (OR 4.50, 95%CI 2.33-8.69; p<0.001), sartans (OR 2.21, 95%CI 1.01-4.80; p=0.046), and CCBs (OR 2.22, 95%CI 1.06-4.67; p=0.034) among concomitant therapies. Pupillometry data instead showed as independent predictors of stroke a high overall mean CH (p<0.001), a slow CV (p=0.011), and high CH and CV absolute differences (p=0.039 and p=0.011, respectively), all with similar ORs as the first model (**Table S3**). However, even in this case, the model did not disclose an overall good fitting. In fact, the analysis of stability and accuracy, though a C-index of 0.903, disclosed a low corrected Dxy rank correlation (0.771 vs. 0.807 of unadjusted Dxy), suggesting the potential low contribution of some other covariates (see also the calibration plot in **Figure S2B**). The related nomogram (see **Figure S4**) suggested us to rule out respiratory disease and overall mean MIN. The final model overall confirmed all the independent predictive factors observed in Model 2, with better significance level. Only sartans did get out and disclosed only a suggestive, though important role (p=0.056) (see **Table S3, Table 4**).
