# Supplementary material for: Automated Pupillometry Is Able to Discriminate Patients with Acute Stroke from Healthy Subjects: An Observational, Cross-Sectional Study
Source: Brain Sci. 2024 Jun 20;14(6):616. doi: 10.3390/brainsci14060616 (PMC11202086; doi:10.3390/brainsci14060616)
Supplement: Supplementary file 1 [file brainsci-14-00616-s001.zip › Supplementary Table S1.pdf]

	Abbreviations	UoM	Definition	ANS branch
**Baseline Pupil Diameter**	BPD	mm	Maximum pupillary diameter at baseline, before the light stimulus	Both
**Minimum Pupil Diameter**	MIN	mm	Pupillary diameter at peak constriction	Parasympathetic
**Reflex Latency**	LAT	s	Time delay between the light stimulus and the onset of pupillary constriction	Parasympathetic
**Average Constriction Velocity**	CV	mm/s	Average velocity of the pupil constriction	Parasympathetic
**Maximum Constriction Velocity**	MCV	mm/s	Maximum velocity of the pupil constriction	Parasympathetic
**%Change – Percentage of Constriction**	CH	%	Obtained by applying the sequent formula: (Size -MIN)/Size*100	Parasympathetic
**Dilation Velocity**	DV	mm/s	Average velocity of the pupil dilation	Both, mostly sympathetic
**Neurological Pupil Index**	NPi	N/A	A composite measure obtained by the combination of all the aforementioned parameters	Both

Table S1 A descriptive table reassuming the parameters collected by NPi-200® (NeurOptics, Irvine, CA, USA). Abbreviations: UoM, Unit of Measurement; ANS, Autonomic Nervous System
