# Supplementary material for: Automated Pupillometry Is Able to Discriminate Patients with Acute Stroke from Healthy Subjects: An Observational, Cross-Sectional Study
Source: Brain Sci. 2024 Jun 20;14(6):616. doi: 10.3390/brainsci14060616 (PMC11202086; doi:10.3390/brainsci14060616)
Supplement: Supplementary file 1 [file brainsci-14-00616-s001.zip › Supplementary Table S2.pdf]

	AIS group (n = 200)	
	**n (%)**	**Median (IQR)**
Pre-admission mRS		0.0 (0.0 - 1.0)
NIHSS at admission		5.0 (2.0 -12.0)
ASPECTs at baseline CT scan		10.0 (9.0 - 10.0)
Intravenous thrombolysis	55 (27.5%)	
Mechanical thrombectomy	66 (33.0%)	
Bridging therapy	25 (12.5%)	
Midline shift ≥ 5 mm at the follow-up neuroradiological examination*	7 (3.5%)	
**TOAST classification**		
Large-artery atherosclerosis	32 (16.0%)	
Cardioembolism	63 (31.5%)	
Small-artery occlusion	36 (18.0%)	
Stroke of other determined etiology	18 (9.0%)	
Stroke of undetermined etiology	51 (25.5%)	
**Stroke side**		
Bilateral/midline strokes	16 (8.0%)	
Left-sided strokes	114 (57.0%)	
**Stroke location**		
Frontal lobe	91 (45.5%)	
Parietal lobe	74 (37.0%)	
Temporal lobe	80 (40.0%)	
Insula	65 (32.5%)	
Occipital lobe	32 (16.0%)	
Basal ganglia	74 (37.0%)	
Thalamus	14 (7.0%)	
Midbrain	9 (4.5%)	
Cerebellum	23 (11.5%)	

Table S2. A summary of the clinical and radiological characteristics of the AIS group. *The time range of the follow-up neuroradiological examination was 12-72 hours after stroke onset. Abbreviations: AIS, Acute Ischemic Stroke; IQR, Interquartile Range; mRS, modified Rankin Scale; NIHSS, National Institute of Health Stroke Scale; ASPECTs, Alberta Stroke Programme Early CT score; CT, Computed Tomography; TOAST, Trial of Org10172 in Acute Stroke Treatment
