# Supplementary material for: Automated Pupillometry Is Able to Discriminate Patients with Acute Stroke from Healthy Subjects: An Observational, Cross-Sectional Study
Source: Brain Sci. 2024 Jun 20;14(6):616. doi: 10.3390/brainsci14060616 (PMC11202086; doi:10.3390/brainsci14060616)
Supplement: Supplementary file 1 [file brainsci-14-00616-s001.zip › Supplementary Table S3.pdf]

	Model 1<br>OR (95%CI); p	Model 2<br>OR (95%CI); p	Model 3<br>OR (95%CI); p
**Demographic data**			
Age	**1.04 (1.02;1.07); 0.001**	**1.05 (1.02;1.07); <0.001**	**1.05 (1.03;1.08); <0.001**
**Comorbidities**			
Diabetes	1.08 (0.45;2.55); 0.865		
Hypertension	1.28 (0.50;3.26); 0.603		
Dyslipidemia	1.58 (0.84;2.94); 0.153		
Previous stroke	**9.05 (2.48;32.95); 0.001**	**10.02 (2.76;36.41); <0.001**	**9.70 (2.69;34.92); 0.001**
Atrial fibrillation	**2.96 (1.04;8.41); 0.042**	**3.37 (1.28;8.87); 0.014**	**3.53 (1.36;9.17); 0.010**
Respiratory disease	2.10 (0.72;6.11); 0.174	2.13 (0.76;5.95); 0.148	
**Pharmacological data**			
Beta-blockers	1.50 (0.70;3.19); 0.294		
Alpha blockers	1.40 (0.49;4.00); 0.533		
ACE-inhibitors	**3.28 (1.40;7.68); 0.006**	**4.50 (2.33;8.69); <0.001**	**4.34 (2.27;8.32); <0.001**
Sartans	1.67 (0.63;4.46); 0.304	**2.21 (1.01;4.80); 0.046**	*2.11 (0.98;4.53); 0.056*
CCBs	1.99 (0.88;4.53); 0.100	**2.22 (1.06;4.67); 0.034**	**2.23 (1.06;4.65); 0.033**
**Pupillometry parameters**			
Overall mean NPi	0.23 (0.01;5.15); 0.355	0.28 (0.01;5.92); 0.416	0.37 (0.10;1.28); 0.116
BPD absolute diff.	*3.10 (0.83;11.49); 0.091*	2.52 (0.73;8.68); 0.143	2.39 (0.73;7.88); 0.151
Overall mean MIN	0.68 (0.07;6.42); 0.740	0.79 (0.88;7.15); 0.854	
Overall mean CH	**1.21 (1.09;1.34); <0.001**	**1.21 (1.10;1.34); <0.001**	**1.21 (1.11;1.33); <0.001**
CH absolute diff.	*1.13 (1.00;1.27); 0.051*	**1.13 (1.01;1.26);0.039**	**1.13 (1.01;1.26); 0.036**
Overall mean CV	**0.30 (0.12;0.75); 0.010**	**0.27 (0.11;0.66); 0.004**	**0.26 (0.11;0.61); 0.002**
CV absolute diff.	*3.90 (0.94;16.14); 0.060*	**4.17 (1.39;12.53); 0.011**	**4.75 (1.64;13.73); 0.004**
MCV absolute diff.	0.90 (0.29;2.78); 0.856		

Table S3 All the three multivariate Logistic Regression Models tested. Abbreviations: M: Males; F: Females; CCBs: Calcium Channel Blockers; NPi: Neurological Pupil index; BPD, Baseline Pupil Diameter; MIN: minimum; CH: Percentage of Constriction; CV: average Constriction Velocity; MCV, Maximum Constriction Velocity; OR: Odd Ratio; CI: Confidence Interval.
