# Supplementary figures and images for: Automated Pupillometry Is Able to Discriminate Patients with Acute Stroke from Healthy Subjects: An Observational, Cross-Sectional Study
Source: Brain Sci. 2024 Jun 20;14(6):616. doi: 10.3390/brainsci14060616 (PMC11202086; doi:10.3390/brainsci14060616)

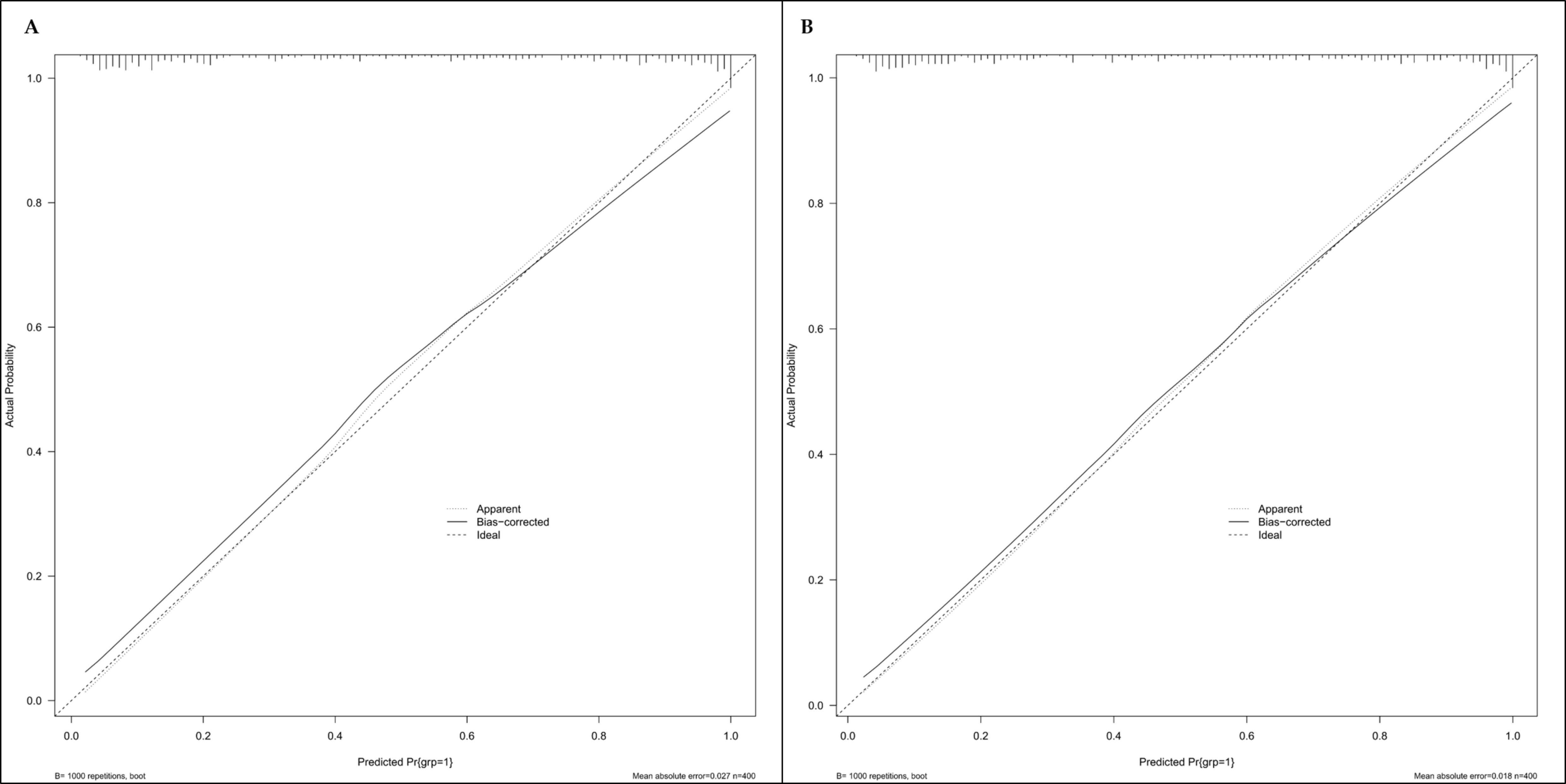

Supplement: Supplementary file 1 [file brainsci-14-00616-s001.zip › Supplementary Figure S2.tiff]

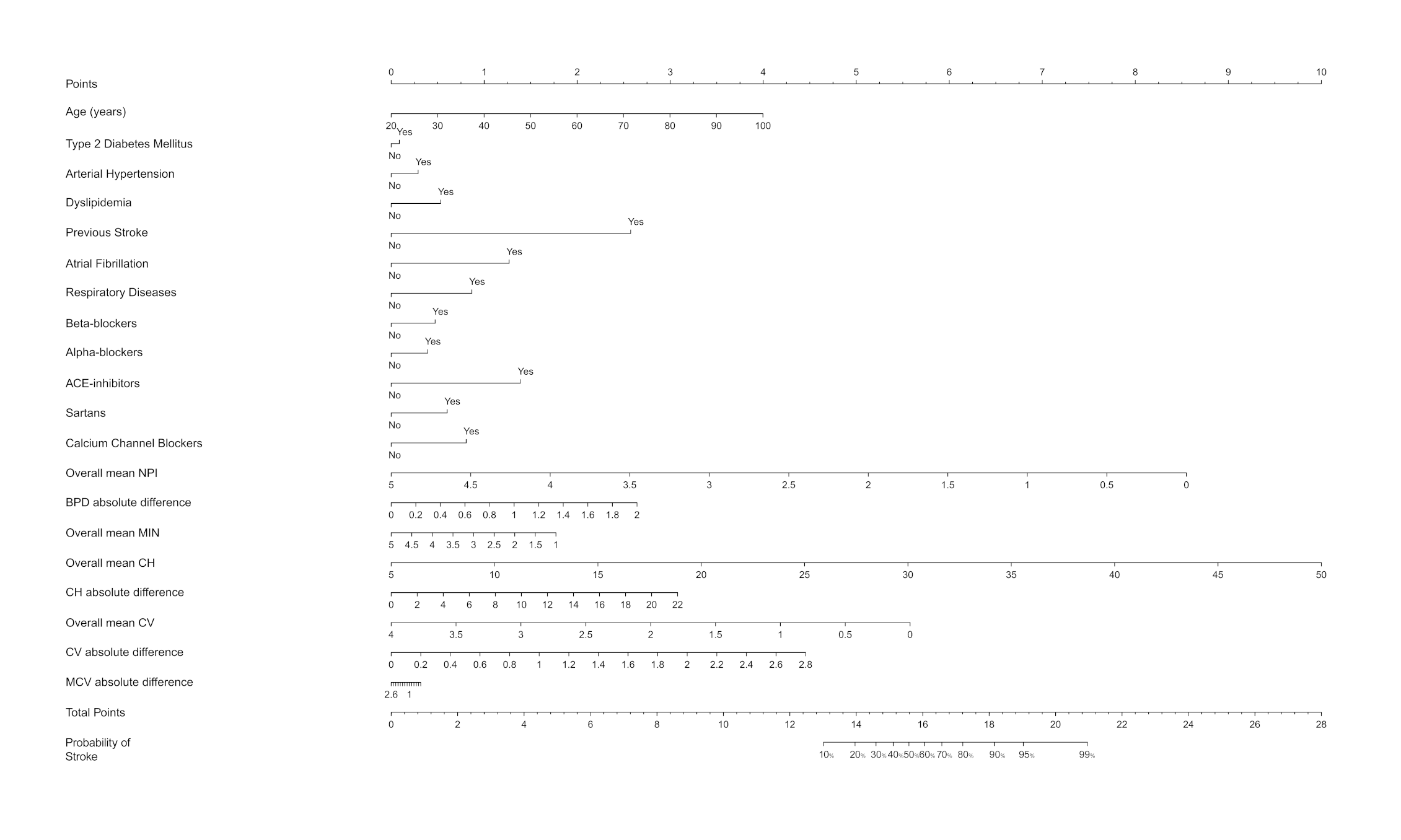

Supplement: Supplementary file 1 [file brainsci-14-00616-s001.zip › Supplementary Figure S3.TIFF]
